# Pre-Existing Anti-Inflammatory Immune Conditions Influence Early Antibody Avidity and Isotype Profile Following Comirnaty^®^ Vaccination in Mice

**DOI:** 10.3390/vaccines13070677

**Published:** 2025-06-24

**Authors:** Mariangeles Castillo, María C. Miraglia, Florencia C. Mansilla, Cecilia P. Randazzo, Leticia V. Bentancor, Teresa Freire, Alejandra V. Capozzo

**Affiliations:** 1 Instituto de Virología e Innovaciones Tecnológicas, Centro de Investigaciones en Ciencias Veterinarias y Agronómicas (CICVyA), INTA-Consejo Nacional de Investigaciones Técnicas (CONICET), Nicolás Repetto & De los Reseros S/N, Hurlingham 1686, Argentina; castillo.mariangeles@inta.gob.ar (M.C.); miraglia.maria@inta.gob.ar (M.C.M.); mansilla.florencia@inta.gob.ar (F.C.M.); randazzo.cecila@inta.gob.ar (C.P.R.); 2 Instituto de Estudios para el Desarrollo Productivo y la Innovación, Universidad Nacional de José Clemente Paz (UNPAZ), Leandro N. Alem 4731, José C Paz, Buenos Aires 1665, Argentina; lbentancor@unpaz.edu.ar; 3 Laboratorio de Inmunomodulación y Vacunas, Unidad Académica Inmunobiología, Facultad de Medicina, Universidad de la República (UdelaR), Avenida General Flores 2125, Montevideo 11800, Uruguay; tfreire@fmed.edu.uy; 4 Centro de Altos Estudios en Ciencias Humanas y de la Salud, CONICET-Universidad Abierta Interamericana, Montes de Oca 745, Buenos Aires 1270, Argentina

**Keywords:** mRNA vaccine, helminth proteins, immunomodulation, trained immunity, early immune response

## Abstract

Background/Objectives: Vaccine immunogenicity is often suboptimal in vulnerable populations such as the elderly, infants, and individuals in low- and middle-income countries. One contributing factor may be pre-existing immunomodulatory conditions, including helminth infections. This study investigates the impact of *Fasciola hepatica* (*F. hepatica*) derived molecules on the early humoral response to the COVID-19 mRNA vaccine Comirnaty^®^ in a mouse model. Methods: BALB/c mice were pretreated with a *F. hepatica* protein extract (FH) or complete Freund’s adjuvant (CFA) prior to vaccination. Cytokine production and antibody responses were assessed at 0, 14, and 21 days post-vaccination (dpv) through serum analysis and ex vivo splenocyte stimulation with the SARS-CoV-2 receptor-binding domain (RBD) or LPS. Results: At 0 dpv, FH-treated mice showed increased serum IL-10, while CFA treatment induced IL-12. FH- but not CFA-treated splenocytes secreted IL-10 upon RBD or LPS stimulation. At 21 dpv, FH-treated mice lacked IFN-γ production but maintained IL-10 and showed elevated IL-4, consistent with a Th2-skewed profile. Although total anti-RBD IgG levels were similar between groups, FH-treated mice exhibited reduced IgG avidity and a higher IgG1/IgG2 ratio. CFA-treated mice showed delayed avidity maturation. Conclusions: Prior exposure to *F. hepatica* antigens can modulate the early immune response to Comirnaty^®^, affecting both cellular activation and antibody quality. This altered response may reflect a reduced early protective capacity of the vaccine, which might need to be considered when designing or evaluating vaccination strategies using mRNA vaccines in helminth-endemic regions.

## 1. Introduction

Infections can profoundly influence trained immunity, a phenomenon where innate immune cells exhibit long-term functional reprogramming in response to stimuli such as infections, vaccines, or adjuvants [1]. Certain infections, particularly chronic or recurrent ones, can either enhance or impair this reprogramming depending on the pathogen and the immune context. For instance, infections that induce immunosuppressive responses, such as those caused by helminths or chronic viral pathogens, may dampen the innate immune system’s capacity to undergo effective training [2]. Conversely, infections that induce robust pro-inflammatory responses might enhance trained immunity but could also risk trigger excessive inflammation, complicating vaccine outcomes [3]. Understanding these interactions is critical for optimizing vaccine efficacy in populations with high burdens of infection, highlighting the need for integrated disease control strategies and the development of vaccines tailored to specific immunological and epidemiological contexts.

The aim of this study was to evaluate whether pre-existing anti- or pro-inflammatory conditions alter vaccine-induced immunity, and to assess their impact on the humoral immune response elicited by the COVID-19 Comirnaty^®^ mRNA vaccine in mice. To achieve this, we employed a *Fasciola hepatica* (*F. hepatica*) protein extract (FH) as an anti-inflammatory regulatory treatment, and Complete Freund’s adjuvant (CFA) which contains inactivated and dried mycobacteria, to create as a pro-inflammatory milieu.

Fasciolosis, caused by the liver flukes *F. hepatica* and *Fasciola gigantica*, is a prevalent neglected tropical disease that affects both humans and animals, particularly livestock; with high impact in public health [4]. It has a significant economic impact due to reduced productivity in animals, including weight loss, decreased milk and meat production, and impaired reproductive performance [5]. In humans, it leads to chronic liver disease, with symptoms ranging from mild abdominal discomfort to severe complications, such as cirrhosis [6]. The disease is more common in rural areas where people live in close contact with livestock, making it a major public health concern. Efforts to control fasciolosis are hindered by limited resources, inadequate diagnostic tools, and insufficient awareness [7] specially in low-and middle-income countries (LMICs) [8].

The immunomodulatory effects of *F. hepatica*, such as downregulation of chemokine receptor expression on macrophages inhibition of macrophage migration [9] and induction of regulatory T cells [10], can impair vaccine-induced immune response. By dampening the host Th1 responses, the parasite can reduce the effectiveness of vaccines that rely on these pathways to generate protective immunity. For instance, vaccines against pathogens requiring robust Th1 responses might not be as efficient as expected in hosts infected with *F. hepatica* due to the parasite-induced suppression of these immune pathways. Some groups have reported that parasite infection alters the induction of antibodies induced by vaccination [11]. However, there are currently no research reports on how the treatment of fasciolosis can affect novel COVID-19 mRNA vaccines.

On the other hand, CFA is a potent immune system enhancer widely used in experimental immunology due to its ability to induce both humoral and cellular immune responses. Its primary mechanism of action is through the activation of the innate immune system, particularly through the stimulation of pattern recognition receptors (PRRs), such as Toll-like receptors (TLRs), which trigger pro-inflammatory cytokine production [12,13]. CFA is also recognized as a promoter of trained immunity, a form of long-lasting functional reprogramming of innate immune cells, leading to an enhanced response to subsequent infections [14,15]. This ability to modulate innate immune responses and induce trained immunity is of particular interest for vaccine development, as it enhances the efficacy of vaccines by boosting the innate immune response long after the initial exposure [1,16].

In this study, we analyzed the humoral immune response in mice vaccinated with the Comirnaty^®^ vaccine following treatment with FH or CFA. These treatments served as models to investigate whether two distinct immune conditions could influence the immune response to a widely used COVID-19 mRNA vaccine, with a special focus on the effect of *F. hepatica* antigens, as this zoonotic neglected tropical parasitic disease is highly prevalent in our region.

## 2. Materials and Methods

### 2.1. Animals and Experimental Design

Female BALB/c mice (five to six-week-old) were purchased from the Faculty of Veterinary Sciences, National University of La Plata (La Plata, Buenos Aires, Argentina). The animals were acclimatized for a week before the beginning of the experiment and kept at the animal facility of CICVyA-INTA (INTA, Hurlingham, Buenos Aires, Argentina) with water and food supplied ad libitum. Mouse handling, care, and experiments were carried out in compliance with institutional guidelines and regulations from the National Committee on Animal Research. Procedures involving animals were approved by the CICUAE Protocol Number 1636649 (request #43/2024 approved on 19 February 2024).

**Figure 1:**
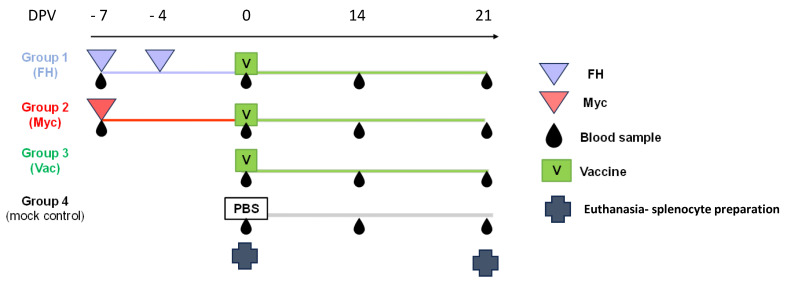
Schematic diagram illustrating the pre-vaccination treatments, vaccination schedule, and corresponding sampling time points.

A total of 49 mice were randomly assigned to four groups. Groups 1, 2, and 4 consisted of 14 animals each, while group 3 consisted of 7 animals. Mice in groups 1 and 2 received different pre-treatments before vaccination, starting one week before vaccination (−7 days post-vaccination, dpv). Mice from group 1 were inoculated intraperitoneally with 100 µL of FH (50 ng/mL) diluted in sterile physiological saline on days −7 and −4 dpv. Mice in group 2 were inoculated subcutaneously at −7 dpv with 100 µL of an emulsion (1:1 in sterile physiological saline) containing 1 mg of heat-inactivated dried *Mycobacterium tuberculosis* (CFA, Sigma-Aldrich Freund’s Adjuvant, Complete F5581) following protocols previously used in mice [17,18]. Groups 3 and 4 received no pretreatment (scheme shown in Figure 1).

Seven animals from groups 1, 2, and 4 were euthanized at 0 dpv, and splenocytes were prepared and cryopreserved following standard protocols [19]. Seven animals from groups 1, 2, and 3 were immunized with 0.1 mL of Omicron BA.4/5 bivalent BNT162b2 Comirnaty^®^ vaccine by intramuscular injection in the left thigh. Serum samples were collected from all animals on −7, 0, 14, and 21 dpv (Figure 1). At 21 dpv, the remaining animals were sacrificed, and splenocytes were cryopreserved for subsequent experiments.

### 2.2. Preparation of F. hepatica Protein Extract (FH)

FH was obtained as previously published [20]. Briefly, *F. hepatica* adult flukes were homogenized in a Teflon homogenizer using PBS in the presence of a protease inhibitor cocktail. The solution was then centrifuged at 40,000× *g* for 60 min at 4 °C, and the protein lysates were dialyzed against PBS. Protein concentration was measured using the bicinchoninic acid assay (Sigma, St. Louis, MO, USA). To remove endotoxin contamination, the extract was applied to a column containing endotoxin-removing gel (Detoxi-gel, Pierce Biotechnology, Waltham, MA, USA), and endotoxin levels were quantified using the Limulus Amebocyte Lysate kit Pyrochrome (Associates of Cape Cod, East Falmouth, MA, USA) and found to be lower than 0.05 EU/mL.

### 2.3. Stimulation of Cryopreserved Splenocytes

Cryopreserved splenocytes were thawed and resuspended in RPMI-1640 medium supplemented with 10% fetal bovine serum (RPMI-10% FBS, ThermoFisher Scientific, Waltham, MA, USA). The cells were incubated at 37 °C in a humidified 5% CO_2_ atmosphere for a few hours to allow recovery. Viable cells from each experimental group were counted and seeded in 96-well U-bottom plates (125,000 cells per well).

Stimulation was performed using either 1 µg/mL of purified recombinant SARS-CoV-2 Spike protein receptor-binding domain (RBD), expressed in *Pichia pastoris* and kindly provided by the Facultad de Ciencias Exactas y Naturales (University of Buenos Aires, UBA) through the Argentine Anti-COVID Consortium [21], or 1 µg/mL of lipopolysaccharide (LPS) from *Escherichia coli* O55:B5 (Sigma-Aldrich, St. Louis, MO, USA). Mock-stimulated cells received PBS, used as the dilution medium.

Cells were incubated for 40 h under standard culture conditions. Plates were then centrifuged at 260× *g* for 5 min, and the supernatants were collected and stored at −80 °C for subsequent ELISA analysis.

### 2.4. Cytokine Quantification by ELISA

Cytokine levels were quantified in cryopreserved supernatants obtained from stimulated splenocyte cultures collected on days 0 and 21 post-vaccination (dpv), as well as in serum samples. Commercial ELISA kits from BD Biosciences Pharmingen were used, including BD OptEIA™ Mouse IFN-γ ELISA Set (Cat. N°.: 555138), Mouse IL-4 ELISA Set (Cat. N°.: 555232), Mouse IL-10 ELISA Set (Cat. N°.: 555252), Mouse IL-12p70 ELISA Set (Cat. N°.: 555256), and Mouse TNF-α ELISA Set II (Cat. N°.: 558534).

The day prior to the assay, ELISA plates were coated with the appropriate capture antibody at a pre-stablished optimal dilution and incubated overnight at 4 °C. On the day of the assay, plates were blocked with PBS containing 10% FBS (Natocor, Córdoba, Argentina) for 1 h at 37 °C. Standard curves (run in duplicate) and test samples were added to the plates and incubated for 2 h at room temperature.

After five washes, detection antibodies and streptavidin–HRP conjugate were added, either as a single incubation step or sequentially, following the manufacturer’s instructions. Following another wash step, the substrate solution (BD OptEIA™ TMB Substrate Reagent Set, Cat. N°.: 555214) was added and the reaction was allowed to develop for 30 min at room temperature in the dark. The reaction was stopped with sulfuric acid, and absorbance was measured at 450 nm using a spectrophotometer (Biotek, Synergy™ HT Reader, Winooski, VT, USA).

### 2.5. Humoral Response Against SARS-CoV-2 RBD

Antibody response against SARS-CoV-2 spike protein RDB were measured following inoculation with Comirnaty^®^ vaccine, adapting a protocol developed in our laboratory using a commercial kit [22]. Briefly, serum was diluted 1:25 with the sample dilution buffer and incubated on the coated microplate for 1 h at room temperature. The wells were washed three times, and then anti-mouse IgG antibody conjugated with HRP (Jackson ImmunoResearch Laboratories Inc., Baltimore, MD, USA) was added and incubated at room temperature for 1 h. For IgG subtype determination anti-IgG1 (BD Pharmingen™ Franklin Lakes, NJ, USA) and anti IgG2 (BD Pharmingen™ Franklin Lakes, NJ, USA) biotinylated antibodies were used, followed by an avidin-peroxidase (KPL Gaithersburg, MD, USA) incubation for 20 min at room temperature.

For avidity determination, serum samples were washed with 6 M Urea for 20 min at room temperature, before following the revealing steps using the anti-mouse IgG antibody. The same samples were washed with PBS. The avidity index (AI%) was estimated as the corrected OD value (cOD, calculated by subtracting blank) of the urea treated sample divided the cOD of the PBS-treated sample multiplied by 100.

The substrate solution was added and incubated at room temperature for 30 min. The stop solution was then added (0.1 mL per well), and absorbance was measured at 450 nm.

### 2.6. Statistical Analysis

Two-way ANOVA followed by Tukey’s multiple comparisons test was used to compare cytokine concentrations between the different groups and treatments and to compare antibody levels between vaccinated groups and the IgG1 to IgG2 ratio at each different time point.

Antibody level whisker plots were created with the median titer and lower and upper range of 5–95%. Correlation and principal component analysis were used to assess total antibody levels avidity IgG, IgG1 and IgG2 levels in the different vaccinated groups. Bland–Altman plots was also used to investigate any possible relationship of the discrepancies between the values obtained after the pre-treatment with FH or CFA and the true value from non-pretreated animals (proportional bias) estimated as the % of the difference as [100 × (non-treated-treated)/average vs. average].

For all the analysis, asterisks indicate statistically significant differences * *p* < 0.05; ** *p* < 0.01; *** *p* < 0.001.

Analysis was performed using GraphPad Prism 10.2.2 (Prism, La Jolla, CA, USA).

## 3. Results

### 3.1. Immune Conditions at the Time of Vaccination

**Figure 2:**
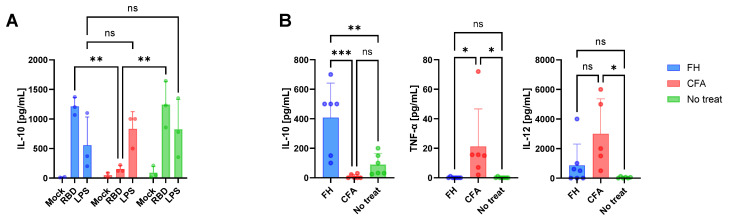
Effects of the in vivo innate training. (**A**) Splenocytes from animals treated in presence or absence of FH or CFA were isolated 7 days after treatment (0 dpv) and stimulated ex vivo with RDB, LPS or PBS (mock). (**B**) Serum cytokine levels in animals treated with FH or CFA at 0 dpv. Concentration of cytokines was estimated from a standard curve using ELISA. Asterisks indicate statistically significant differences between groups calculated with two-way Anova followed by a Tukey multiple comparison test: * *p* < 0.05; ** *p* < 0.01; *** *p* < 0.001; ns = not significant (*p* > 0.05).

Splenocytes from treated and untreated mice were stimulated ex vivo RBD or LPS to examine the immune status at the time of vaccination (Figure 2A). In untreated mice, IL-10 production in response to RBD and LPS was moderate. In contrast, splenocytes from FH-treated animals secreted significantly higher levels of IL-10 in response to both stimuli. In the CFA group, IL-10 production was not detected following RBD stimulation but was produced after LPS exposure.

Inoculation with either FH or CFA resulted in distinct serum cytokine profiles for IL-10, TNF-α, and IL-12 at 0 dpv (Figure 2B). Although additional cytokines, including IL-4 and IFN-γ, were assessed, their levels were undetectable. Administration of two doses of FH induced a cytokine milieu characterized by elevated IL-10 levels compared to untreated or CFA-treated mice. Conversely, TNF-α levels were significantly higher in the CFA-treated group than in the other groups. Similarly, CFA treatment elicited higher IL-12 levels compared to FH-treated or untreated groups.

These findings indicate that FH inoculation predominantly elicited an innate regulatory anti-inflammatory response, while CFA administration induces a pro-inflammatory innate immune response. No distinctive cytokine profile was observed in untreated animals.

### 3.2. Vaccine-Induced Humoral and Cytokine Immune Responses

**Figure 3:**
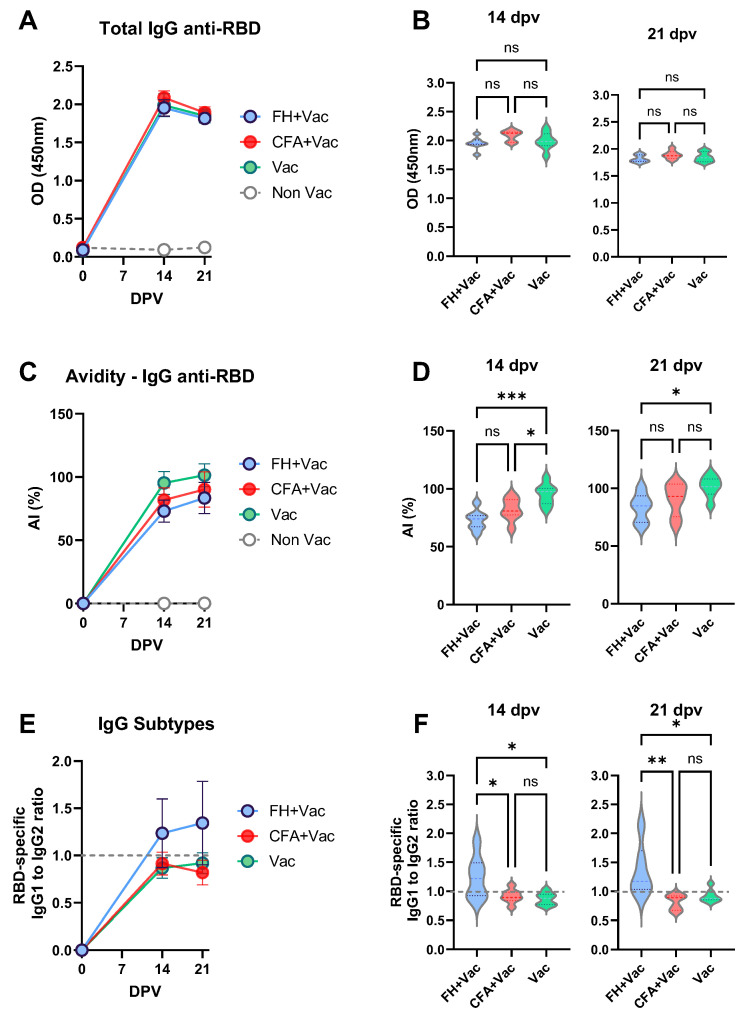
RBD-specific antibody immune response in *F. hepatica* treated (blue), CFA-treated (orange) or control (green) mice. (**A**) Total anti-RBD IgG kinetics and (**B**) compared levels at 14 and 21 dpv. (**C**) Kinetics of the avidity maturation expressed as avidity index (AI%) and (**D**) compared levels at 14 and 21 dpv. (**E**) IgG1 to IgG2 ratio kinetics, and (**F**) comparisons at 14 and 21 dpv with line dotted line indicating equal concentration of both IgG subtypes. Asterisks indicate statistically significant differences between infected and control animals calculated with two-way Anova followed by a Tukey multiple comparison test: * *p* < 0.05; ** *p* < 0.01; *** *p* < 0.001; ns = not significant (*p* > 0.05).

The potential modulation of vaccine-induced immunity by FH and CFA treatments was examined by analyzing the humoral response to Comirnaty^®^ vaccination. Total IgG, IgG avidity, and IgG isotypes specific to the RBD were measured across all groups (Figure 3). The kinetics of RBD-specific total IgG were comparable among vaccinated mice, with similar antibody levels detected at both 14- and 21- dpv (Figure 3A,B). Cumulative IgG responses from 0 to 21 dpv were assessed by calculating the area under the curve (AUC) for each group, revealing no significant differences between pre-treated and untreated mice (Table 1). Although a slight decrease in IgG levels was noted at 21 dpv, relative to 14 dpv, across all groups, this reduction was not statistically significant (*p* < 0.05) and was consistent with typical fluctuations in antibody plateau levels.

Significant differences emerged when analyzing the avidity index of RBD-specific IgG, an indirect measure of immune response maturation and antibody quality [23,24]. The AUC analysis revealed that untreated mice produced antibodies with higher avidity compared to the other groups, with a statistically significant reduction for the FH-treated group (Table 1). In untreated vaccinated mice, RBD-specific IgG avidity increased rapidly by 14 dpv and continued to rise at 21 dpv (Figure 3C,D). Although a similar pattern was observed in the FH- and CFA-treated groups, their overall IgG avidity levels were lower. Mice pre-treated with FH before vaccination produced RBD-specific IgG with lower avidity compared to untreated mice both at 14 and 21 dpv (Figure 3D), indicating that *F. hepatica*-derived molecules influenced the quality of the antibody response induced by the mRNA vaccine. This was further supported by Bland–Altman analysis, which demonstrated that mean avidity index (AI) values for FH-treated mice were 26% lower than those of untreated mice at 14 dpv and 19% lower at 21 dpv.

The inflammatory environment at the time of vaccination also affected the IgG subtype profile. Pre-treatment with FH shifted the immune response toward an IgG1-dominated profile, with an IgG1:IgG2 ratio exceeding 1 at both time points (Figure 3E,F). In contrast, untreated vaccinated mice exhibited higher IgG2 levels (Figure 3F), as indicated by the AUC analysis (Table 1).

**Figure 4:**
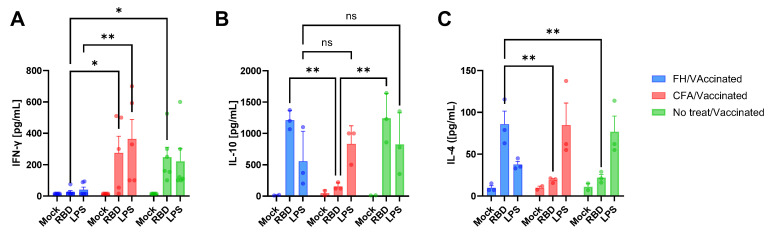
Splenocytes from vaccinated animals pre-treated, or not pre-treated, with FH or CFA and vaccinated with one dose of Comirnaty^®^ Vaccine (legends, “Animals”) were isolated at 21 dpv. Cells were stimulated ex vivo (indicated by “Cells”) with RBD, LPS or mock-treated and IFN-γ (**A**), IL-10 (**B**) and IL-4 (**C**) concentrations were estimated by ELISA. * *p* < 0.05; ** *p* < 0.01; ns = not significant (*p* > 0.05).

The impact of pre-existing immune conditions on vaccine response was investigated further by measuring cytokines following ex vivo stimulation of splenocytes (21 dpv) with RBD or LPS (Figure 4). Splenocytes from untreated vaccinated animals produced high levels of IFN-γ, IL-10, but low levels of IL-4 (Figure 4A,B and Figure 4C, respectively) in response to either stimulus. In contrast, CFA-treated animals produced high levels of IFN-γ following RBD stimulation (Figure 4A) and lower levels of IL-4 (Figure 4C) compared to FH-treated animals, and lower levels of IL-10 (Figure 4B) compared to all the other groups, indicating that this treatment based the immune response towards a Th1 response. FH-treated animals displayed a distinct cytokine profile, with elevated IL-10 and IL-4 levels and suppressed IFN-γ production following RBD stimulation. These splenocytes were less responsive to LPS stimulation, producing lower levels of cytokines than the other groups.

The pro-inflammatory treatment (CFA) did not alter the cytokine response to RBD compared to non-treated animals, except for IL-10 levels, and IgG isotype responses were similar, although avidity maturation was delayed. In contrast, *F. hepatica*-derived components promoted a regulatory environment, characterized by reduced antibody avidity and a predominance of IgG2 over IgG1. These findings highlight the differential impact of pro- and anti-inflammatory milieu at the time of vaccination on the humoral and cytokine responses elicited by the Comirnaty^®^ vaccine.

## 4. Discussion

Skewing of the innate immune response at the time of vaccination can affect the magnitude and quality of vaccine-induced adaptive responses, with either beneficial or detrimental outcomes depending on the immunological context. Although there is increasing interest in the modulatory effects of the inflammatory environment on vaccine efficacy, data regarding how pro- or anti-inflammatory conditions influence responses to mRNA-based vaccines remain limited. In this study, we specifically evaluated the antibody and cytokine responses elicited by mRNA vaccination under a defined inflammatory milieu at the time of immunization, with a special focus on *F. hepatica* antigens.

We successfully established distinct pro- and anti-inflammatory conditions in vivo, which were clearly defined at the time of vaccination. As expected, Complete Freund’s Adjuvant induced elevated levels of IL-12 and TNF-α, consistent with its well-characterized pro-inflammatory properties. CFA administration in mice activates antigen-presenting cells and promotes the production of pro-inflammatory cytokines, including TNF-α [25].

Conversely, we verified the presence of high levels of IL-10 after FH treatment at 0 dpv, which could also be detected in sera at the time of vaccination, while pro-inflammatory cytokines were not produced, consistent with bibliography data regarding immune cells exposed to *F. hepatica* or *F. hepatica*-derived proteins [2]. Previous studies have demonstrated that *F. hepatica* cathelicidin-like proteins [26,27,28] possess immunoregulatory properties that can affect the function of dendritic cells and macrophages [29], inducing regulatory dendritic cells [30,31,32,33] and alternative activated macrophages [34,35].

IL-10 also has a role during SARS-CoV-2 infection. A study in rhesus macaques revealed that IL-10 plays a dual role: it suppresses the expansion of T cells while promoting the formation of tissue-resident memory T cells in the airways. This suggests that IL-10 modulates the immune response, balancing between limiting inflammation and facilitating long-term immunity [36]. Our findings reinforce the role of *F. hepatica* and CFA treatment in modifying this equilibrium, with the latter completely blocking IL-10 production at the time of vaccination, potentially affecting long-term immunity and promoting tissue damage.

We also observed significantly increased IL-10 levels in response to RBD stimulation before vaccination, both in serum and splenocytes compared to untreated or CFA-treated cells. This result aligns with a study reporting that the SARS-CoV-2 RBD can directly promote IL-10 secretion in splenocytes [37]. The presence of high levels of IL-10 can interfere with the development of robust, protective immune responses which may explain, at least in part, the need of revaccinations to maintain immunity against SARS-CoV-2. More studies are needed to investigate this hypothesis.

Our data revealed no differences in seroconversion rates between groups regardless of the inflammatory conditions at the time of vaccination. However, qualitative differences in the antibody response were observed, particularly in IgG isotype distribution and antibody avidity.

The inflammatory milieu promoted by CFA likely promoted the differentiation of T helper 1 (Th1) response cells as expected [12]. This is supported by the higher systemic levels of IgG2 compared to IgG1 and by the production of pro-inflammatory cytokines by splenocytes upon RBD stimulation. The absence of IL-10 further reflects the excessive inflammatory environment induced by CFA, as IL-10 plays a key role in limiting inflammation and maintaining immune homeostasis, which was likely disrupted by the treatment [38]. Recent findings also suggest that serum metabolite profiles altered by CFA may contribute to immune modulation and affect the immune homeostatic balance [39].

Despite this Th1-skewed environment, the effect of CFA on antibody avidity was limited to the initial response following the first vaccine dose, possibly due to an insufficient or transient pro-inflammatory bias that diminished as the immune response matured. Further studies with extended sampling and more detailed analysis are needed to evaluate the persistence of this effect. In contrast, treatment with *F. hepatica* extract resulted in significantly lower IgG avidity at both 14 and 21 days post-vaccination, compared to untreated controls. This finding suggests a marked delay in immune maturation and highlights the immunomodulatory capacity of helminth-derived proteins, which may interfere with the development of high-affinity antibodies in response to mRNA-based COVID-19 vaccination.

The impact of the pro- or anti-inflammatory condition at the time of vaccination on the avidity maturation of the antibodies might be relevant for the efficacy COVID-19 vaccines. Multiple studies have shown that antibody avidity, rather than neutralizing antibody levels, is associated with the severity of COVID-19 and clinical outcomes [40,41]. This suggests that antibody avidity contributes significantly to protection, particularly when high-affinity antibodies recognize key protective epitopes, like those in RBD measured in this study.

The immune modulation caused by *F. hepatica* proteins we described here is similar to that observed for other vaccines, predisposing to an adaptive immune response characterized by T-helper type 2 (Th2) and regulatory T cell (Treg)-associated cytokines [11,26,35,42,43,44]. The isotype profile of the IgG (higher IgG1 than IgG2 levels) elicited together with the production of high levels of IL-10 and IL-4 after exposure to RBD ex vivo, suggest a mixed Th2-regulatory skewed specific response to RBD promoted by FH. It has been reported that the protein extract we used in this study induces DC modulation, provoking the absence of T-cell Th1 cytokine response and proliferative activity [10]. A previous work from our group demonstrated that *F. hepatica* infection alters antibody quality induced by a commercial Foot-and-Mouth Disease vaccine in cattle [11].

Our primary research focus is investigating how *F. hepatica* antigens modulate immune responses to vaccines, with particular attention paid to mRNA platforms. Previous studies in outbred cattle, conducted in our laboratory [11,45], have shown that exposure to *F. hepatica* can dampen the efficacy of both viral and bacterial inactivated vaccines, even in the presence of pro-inflammatory adjuvants. These findings led us to question whether a similar immunoregulatory effect could be observed with mRNA vaccines, such as Comirnaty, which is currently widely administered across our region.

BALB/c mice were chosen because they provide a well-characterized model for *F. hepatica*-induced immunomodulation. This strain is known to exhibit a Th2-prone phenotype, which synergizes with the regulatory and Th2-biased immune environment elicited by *F. hepatica* antigens, including increased IL-10 production and expansion of regulatory T cells [9,10]. Studies on the immunological effects of *F. hepatica* and other helminths have used BALB/c mice as a reference model for this reason [8,43]. The use of this strain thus provided a consistent and reproducible system to address our central hypothesis.

We acknowledge the importance of mouse strain-dependent (Th1/2) differences in immune responses, including to mRNA vaccines [46,47], and would be the focus of future studies. A recently published commentary by L. Chacín-Bonilla [48] discussed how a Th2-skewed cytokine response in individuals infected with helminths may either enhance or impair the course of COVID-19 [49,50,51,52]. However, the effect of vaccines was not analyzed. In this regard, experimental studies such as ours provide important evidence on the role of *F. hepatica* in modulating the immune response to COVID-19 mRNA vaccines.

## 5. Conclusions

The results obtained in this work indicate that anti-inflammatory pre-existing conditions alter vaccine-induced immunity and, therefore, highlight the necessity to determine modifications in how vaccine efficacy against COVID-19 is assessed in humans, particularly within specific healthcare settings in low and middle-income countries (LMICs), where neglected tropical parasitic infections like fasciolosis are widespread.

## Figures and Tables

**Table 1:** Evaluation of humoral immune parameters against the RBD, including total specific IgG, avidity, and IgG1 and IgG2 levels. The total area under the curve (AUC) of the kinetic response is presented along with the 69% confidence intervals. * Significantly higher than Group 1 (*p* < 0.05).

Parameter	AUC	Group 1 (FH/ Vaccinated)	Group 2 (Myc/ Vaccinated)	Group 3 (No Treatment/ Vaccinated)	Group 4 (Not Vaccinated)
Total IgG	Total Area	27.50	29.37	27.95	2.24
	95% Confidence Interval	25.74 to 29.25	27.82 to 30.92	27.82 to 30.92	0.4 to 1.66
IgG avidity	Total Area	1060	1176	1356 *	2.10
	95% Confidence Interval	901.6 to 1202	1000 to 1352	1213 to 1509	0.33 to 1.45
IgG1	Total Area	7.69	7.11	8.25	2.25
	95% Confidence Interval	6.71 to 8.66	5.08 to 9.14	7.11 to 9.38	1.44 to 3.05
IgG2	Total Area	6.60	8.97	9.35 *	1.89
	95% Confidence Interval	4.65 to 8.06	7.86 to 10.08	8.27 to 10.42	1.58 to 2.2

## Data Availability

The data that support the findings of this study are available from the corresponding author upon request.
